# Sunscreen use and intentional exposure to ultraviolet A and B radiation: a double blind randomized trial using personal dosimeters

**DOI:** 10.1054/bjoc.2000.1429

**Published:** 2000-11

**Authors:** P Autier, J-F Doré, A C Reis, A Grivegnée, L Ollivaud, F Truchetet, E Chamoun, N Rotmensz, G Severi, J-P Césarini

**Affiliations:** Division of Epidemiology and Biostatistics, European Institute of Oncology, Milan, Italy; INSERM Unit 453, Centre Léon Bérard, Lyon, France; , Joint Engineering Group, Brussels, Belgium; Unit of Epidemiology and Prevention, J Bordet Institute, Brussels, Belgium; Department of Dermatology (Pr. L. Dubertret), Hopital Saint Louis, Paris, France; Department of Dermatology, Hopital Beauregard, Centre Hospitalier Regional de Metz-Thionville, France; INSERM – L.R.P.T.H., Fondation A. de Rothschild, Paris, France

**Keywords:** ultraviolet radiation, sunscreen, randomized trial, skin cancer

## Abstract

A previous randomized trial found that sunscreen use could extend intentional sun exposure, thereby possibly increasing the risk of cutaneous melanoma. In a similarly designed trial, we examined the effect of the use of sunscreens having different sun protection factor (SPF) on actual exposure to ultraviolet B (UVB) and ultraviolet A (UVA) radiation. In June 1998, 58 European participants 18–24 years old were randomized to receive a SPF 10 or 30 sunscreens and were asked to complete daily records of their sun exposure during their summer holidays of whom 44 utilized a personal UVA and UVB dosimeter in a standard way during their sunbathing sessions. The median daily sunbathing duration was 2.4 hours in the SPF 10 group and 3.0 hours in the SPF 30 group (*P* = 0.054). The increase in daily sunbathing duration was paralleled by an increase in daily UVB exposure, but not by changes in UVA or UVB accumulated over all sunbathing sessions, or in daily UVA exposure. Of all participants, those who used the SPF 30 sunscreen and had no sunburn spent the highest number of hours in sunbathing activities. Differences between the two SPF groups in total number of sunbathing hours, daily sunbathing duration, and daily UVB exposure were largest among participants without sunburn during holidays. Among those with sunburn, the differences between the two groups tended to reduce. In conclusion, sunscreens used during sunbathing tended to increase the duration of exposures to doses of ultraviolet radiation below the sunburn threshold. © 2000 Cancer Research Campaign


*British Journal of Cancer* (2000) **83**(9), 1243–1248
© 2000 Cancer Research Campaign
doi: 10.1054/ bjoc.2000.1429, available online at http://www.idealibrary.com on IDEAL®

### Sunscreen use and intentional exposure to ultraviolet A and B radiation: a double blind randomized trial using personal dosimeters

**P Autier[1], J-F Doré[2], AC Reis[3], A Grivegnée[4], L Ollivaud[5], F Truchetet[6], E Chamoun[4], N Rotmensz[1], G Severi[1], J-P Césarini[7] for the EORTC* Melanoma Co-operative Group**

[1]Division of Epidemiology and Biostatistics, European Institute of Oncology, Milan, Italy; [2]INSERM Unit 453, Centre Léon Bérard, Lyon, France; [3]Joint Engineering Group, Brussels, Belgium; [4]Unit of Epidemiology and Prevention, J Bordet Institute, Brussels, Belgium; [5]Department of Dermatology (Pr. L. Dubertret), Hopital Saint Louis, Paris, France; [6]Department of Dermatology, Hopital Beauregard, Centre Hospitalier Regional de Metz-Thionville, France; [7]INSERM – L.R.P.T.H., Fondation A. de Rothschild, Paris, France

**Summary** A previous randomized trial found that sunscreen use could extend intentional sun exposure, thereby possibly increasing the risk of cutaneous melanoma. In a similarly designed trial, we examined the effect of the use of sunscreens having different sun protection factor (SPF) on actual exposure to ultraviolet B (UVB) and ultraviolet A (UVA) radiation. In June 1998, 58 European participants 18–24 years old were randomized to receive a SPF 10 or 30 sunscreens and were asked to complete daily records of their sun exposure during their summer holidays of whom 44 utilized a personal UVA and UVB dosimeter in a standard way during their sunbathing sessions. The median daily sunbathing duration was 2.4 hours in the SPF 10 group and 3.0 hours in the SPF 30 group ($P$ = 0.054). The increase in daily sunbathing duration was paralleled by an increase in daily UVB exposure, but not by changes in UVA or UVB accumulated over all sunbathing sessions, or in daily UVA exposure. Of all participants, those who used the SPF 30 sunscreen and had no sunburn spent the highest number of hours in sunbathing activities. Differences between the two SPF groups in total number of sunbathing hours, daily sunbathing duration, and daily UVB exposure were largest among participants without sunburn during holidays. Among those with sunburn, the differences between the two groups tended to reduce. In conclusion, sunscreens used during sunbathing tended to increase the duration of exposures to doses of ultraviolet radiation below the sunburn threshold. © 2000 Cancer Research Campaign

**Keywords:** ultraviolet radiation; sunscreen; randomized trial; skin cancer

Recreational, intermittent sun exposure is believed to be the main environmental risk factor for cutaneous melanoma (hereafter referred as ‘melanoma’) (IARC, 1992; Elwood and Jopson, 1997). If ultraviolet radiation (UV) seems to represent the part of the solar spectrum involved in melanoma occurrence, the respective role of ultraviolet B (UVB, 280–315 nm) and ultraviolet A (UVA, 315–400 nm) radiation in melanoma genesis is still unknown (Setlow and Woodhead, 1994; Donawho and Wolf, 1996).

During the summer 1997, in a double-blind randomized trial with European participants aged 18 to 24 years old, we demonstrated that use of high sun protection factor (SPF) sunscreen could lead to longer stays in the sun (Autier et al, 1999). The SPF is a measure of the ability of a sunscreen product to delay sun-induced sunburns. That phenomenon may explain why in epidemiological studies, sunscreen use during sunbathing was often associated with a higher risk of melanoma, basal cell skin cancer or higher nevi count (Bigby, 1999; Autier, 2000). However it is not known which aspect of prolonged sun exposure is responsible for the increased melanoma risk, e.g. UVA exposure (Lui et al, 1997).

In this study, which reproduced the double-blind randomized design of summer 1997, we used personal UV-dosimeters to examine how, in the usual conditions of their use sunscreens of different SPF would influence exposure to UVB and UVA radiation.

This study focused on sunbathing since, as shown during the 1997 trial, use of higher SPF sunscreen led to longer sunbathing (Autier et al, 1999). Also, the average European adult vacationer more often uses sunscreens for sunbathing than for other outdoor activities. Moreover sunbathing is typical of the intermittent sun exposure pattern, and a known risk factor for cutaneous melanoma in white European populations (Autier et al, 1994; Osterlind et al, 1988).

### METHODS

The personal UV-dosimeters used in this trial were genuine prototypes. Because of their cost, only 50 personal dosimeters were available, and we could therefore not conduct a trial with a sample size as large as during the summer 1997 when 86 participants were randomized. 12 participants without personal UV-dosimeter were nonetheless further recruited in order to increase statistical power, so that we could determine whether the previous findings could be reproduced with participants originating from different environments and having vacations in different places.

*Received 29 February 2000*
*Revised 23 June 2000*
*Accepted 25 June 2000*

*Correspondence to:* G Severi

*EORTC is the acronym for the European Organisation for Research and Treatment of Cancer.

### Trial design

The design, randomization method, questionnaires, commercial sunscreens used, sunscreen repackaging, selection and informing of participants strictly followed the summer 1997 trial design (Autier et al, 1999). In brief, in June 1998, 62 Belgian and French paid participants 18–24 years old were randomized to receive a SPF 10 or a SPF 30 sunscreen (320 g per volunteer). Participants were asked to record their sun exposure in standard record diaries on a daily basis. In order to keep the trial as close as possible to habitual conditions of sunbathing, no specific recommendation was made about sun exposure or sunscreen use. The stated trial endpoint for participants and medical personal who had direct contacts with them was the number of pigmented skin lesions before and after the holidays.

The trial was implemented in three institutions not involved in the summer 1997 trial: the St Louis hospital (Paris, France, 16 participants randomized), the Beauregard hospital (Thionville, a town located in the North of France, 16 participants randomized), and the Jules Bordet Institute (Brussels, Belgium, 30 participants randomized). The study was conducted in accordance with the principles of the Helsinki declaration, and was submitted for approval to the Ethical Review Committee of each participating institution. Each volunteer signed a written informed consent before randomization.

### Personal dosimeters

The dosimeter dimensions were 9.5 × 6.0 × 2.5 cm. It comprised on its largest surface a UVB and a UVA captor, able to record 100% radiation within an angle of 60 degrees (Laser Components GmbH, Olching, Germany). The UVA captor had its maximal sensitivity for the 340 nm wavelength, and had a sensitivity ≥ 10% of the maximal sensitivity from 315 to 395 nm. The UVB captor had its maximal sensitivity for the 270 nm wavelength, and had a sensitivity ≥ 10% of the maximal sensitivity from 240 to 310 nm. These captors were heat stable and water-resistant (but not waterproof). Every 20 minutes, the recorded UVB and UVA were stored in the memory. UV recording was automatic and permanent. Participants had not to manoeuvre any button to start the dosimeter. A lithium battery ensured 3-month power autonomy. Time recording corresponded to the so-called Continental summer hour, equivalent to the solar hour plus 2 hours. A port inserted in the dosimeter allowed data transfer to a computer database using custom software.

The dosimeters were calibrated using recommendations from the American Conference of Governmental Industrial Hygienists issued in 1997. Details on calibration procedure may be obtained from the authors.

### Random allocation of dosimeters and instruction to participants

Personal dosimeters were given to participants during the initial visit in June 1998, on the basis of ‘first come – first served’. Participants received standard instructions on how to use the dosimeter: when sunbathing, they had to install the dosimeter nearby them in the sunlight with captors oriented horizontally to the ground.

### Data analysis

For each day, a question in the standard record diaries specifically inquired about the type of sun exposure that took place at a given moment of the day. Each time a volunteer reported a sunbathing activity, he or she had to indicate the starting and ending hour. Sunbathing duration was derived from the daily record diaries. UV exposure was derived from the dosimeters.

Sun induced erythemal reaction is essentially due to the UVB radiation. Throughout the analysis, UVB exposure is expressed in terms of minimal erythemal dose (MED, with 1 MED being equivalent to 200 J/m$^2$). One MED is the lowest radiant exposure to UVR that is sufficient to produce erythema with sharp margins 24 hours after exposure (IARC, 1992). In this trial, a skin erythema had to be painful for being considered as sunburn. Depending on individual sun sensitivity, sunburn occurs after UV exposure equivalent to 2 to 4 MEDs.

The distribution of sun exposure duration and UV exposure, being skewed to the right, departed from Gaussian distribution. Also, during analysis, small numbers of participants were encountered in some strata. Therefore, when not otherwise specified, medians and inter quartile ranges were used for presenting summary statistics. In the latter case, non-parametric tests were used for testing differences in data distribution between the two randomization groups. When means were used as summary statistics, the student *t*-test or the analysis of variance methods were used to test differences. Least-squares regression multivariate analysis was used to assess the influence of different factors on study end points, after log transformation of endpoints. All statistical tests were two-sided.

### RESULTS

The randomization process allocated 31 participants in the SPF 10 group and 31 participants in the SPF 30 group. One volunteer in the SPF 10 group (without dosimeter) had a car accident when going on holiday, and could not take part in the trial. Three participants, one in the SPF 10 arm (who had a dosimeter) and two in the SPF 30 arm (who had a dosimeter), did not sunbathe during their holidays, because of bad weather or sickness. Hence, for these four participants, no data on sunbathing and related UV exposure could be taken into account.

Distributions by sex, study place and skin phototypes were equivalent in both randomization arms (Table 1). Fifty-five per cent of areas where SPF 10 participants spent their holidays were located on the Mediterranean coast, 13% in tropical or subtropical zones, and 32% were in the countryside or on the North Sea coast. These proportions were 60, 10 and 30% for SPF 30 participants, respectively.

Mean (range) use of sunscreens by participants was 67 (13–128) g in the SPF 10 group and 77 (2–178) g in the SPF 30 group (Student *t*-test $P = 0.22$). A sunscreen other than the one offered was used during 5% and 6% of days with sun exposure in the SPF 10 and SPF 30 group, respectively ($\chi^2$ test: $P > 0.50$). History of sunburns during holidays was equivalent in the two groups (Table 1).

The dosimeters of 6 participants did not provide relevant information (Table 1), so UV recordings were informative for 23 SPF 10 and 21 SPF 30 participants. The distribution of skin phototype was equivalent in the two groups of participants with a dosimeter and carrying a dosimeter seemed not to have affected either sunburn experience or quantities of sunscreen used (data not shown). In both groups, participants used the dosimeter during

 © 2000 Cancer Research Campaign

**Table 1** Characteristics of participants

| | SPF 10[a] (*n* = 29) | SPF 30 (*n* = 29) |
|---|---|---|
| French, Paris | 8 | 7 |
| French, Thionville | 7 | 7 |
| Belgian, Brussels | 14 | 15 |
| Females | 20 | 23 |
| Males | 9 | 6 |
| Skin Phototype[b] | | |
| I | 1 | 2 |
| II | 15 | 16 |
| III | 13 | 11 |
| Number of participants with | | |
| No sunburn episode during the trial | 12 | 11[c] |
| 1 sunburn episode during the trial | 10 | 7 |
| ≥ 2 sunburn episodes during the trial | 7 | 11 |
| Participants who received a dosimeter | 26 | 24 |
| Participants with informative dosimeter | 23 | 21 |
| Did not sunbathe | 1 | 2 |
| Did not use the dosimeter | 0 | 1 |
| Defective dosimeter | 2 | 0 |

[a] SPF: Sun Protection Factor; [b] When going in the sun, skin phototype I always burns and never tans; skin phototype II always burns first and tans after; skin phototype III sometimes burns but always get a deep tan (Fitzpatrick, 1988); [c] $\chi^2$ of heterogeneity: $P = 0.46$.

three-quarters of all days with sunbathing: sometimes they forgot to bring the dosimeter to the beach, or they sunbathed in swimming pools, and preferred not to use the dosimeter because of the proximity of the water. There was no indication in the diaries that amounts of UV exposure were different during days with or without use of the dosimeter.

Use of the SPF 30 sunscreen was associated with a 25% increase in duration of daily sunbathing (Table 2). A slightly larger difference in duration was noticeable for sunbathing activities before 6 PM (i.e., 4 PM solar hour). In order to control for potential confounding effects, we fitted a linear regression model with daily sunbathing duration as dependant variable. The SPF (10 versus 30), the in-trial sunburn experience (none versus ≥ 1), the skin phototype (I–II versus III), and the quantity of sunscreen used (continuous variable) were the independent variables. The adjusted extra time spent in sunbathing activities when the SPF 30 sunscreen was used was 33 minutes (95% confidence interval: 3 to 63 minutes, $P = 0.020$).

The starting hour of sunbathing activities was identical in both groups during the first two holiday days with sunbathing (Figure 1). As holidays progressed, however, SPF 30 participants tended to start sunbathing systematically earlier in the day, while SPF 10 participants tended to start sunbathing systematically later in the day. As a consequence, sunbathing after 6 PM was more prevalent among SPF 10 participants (Table 2), who were apparently more inclined to take advantage of the sunniest days for sunbathing longer during the late afternoons and evenings.

Differences in the timing of sunbathing between the two groups (Table 2 and Figure 1) had effects on UVA and UVB exposure: according to dosimeters (Figure 2), most intense exposures before 2 PM found among SPF 30 participants. After 4 PM SPF 10 participants had more intense exposure to UV, particularly UVA. In both groups, UVA and UVB received by participants did not parallel typical proportions of UVA and UVB reaching earth's surface over the day in a Mediterranean area (shown in the lower part of Figure 2).

The total UVA and UVB exposure accumulated during all sunbathing sessions were quite similar in both groups (Table 2), but with substantial interindividual variation. Taking 1 MED equivalent to 200 J/m$^2$ of UVB (IARC, 1992), sunbathing activities during summer 1998 holidays were associated with an average accumulated UVB exposure of ~42 MEDs after correction for days without dosimeter.

Data in Table 2 show that it was the increase in median UVB exposure per day with sunbathing that best paralleled the increase in daily sunbathing duration of SPF 30 participants. No, or smaller difference emerged between the two groups for accumulated and daily UVA exposure.

In epidemiological studies, the largest difference in melanoma risk (or in nevi count) between sunscreen users and non-users was found among subjects without sunburn history (Autier et al, 1995, 1998; Westerdahl et al, 2000). We therefore analysed data according to sunburn experience during the trial (Table 3). The largest differences in total number of sunbathing hours on holidays, and in daily sunbathing duration was observed among participants who did not suffer from sunburn on holidays. These differences were statistically significant in spite of the small number of participants in each category of sunburn by SPF group. SPF 30 participants without sunburn spent the highest number of hours in sunbathing activities. When sunburn was experienced during holidays, the total and daily sunbathing duration tended to become equivalent in the two groups. In a linear regression model with the total number of sunbathing hours as endpoint, a positive interaction with an associated *P* value of 0.08 became apparent between use of a SPF 30 sunscreen and the absence of sunburn.

Amounts and differences in accumulated UVB over holidays did not correlate, whereas daily UVB exposure correlated well, with sunburn experience (Table 3). In the SPF 10 group, a median daily UVB dose of 511 J/m$^2$ (i.e., ~2.5 MEDs) had no consequence in terms of sunburn. An increase in sunbathing duration of 0.7 hours per day, representing a median extra daily UVB exposure of ~1.5 MEDs, was associated with one sunburn episode in the SPF 10 group, but not in the SPF 30 group. Participants in the latter group had to accrue a supplementary UVB dose of ~ 1MED before getting a sunburn, indicating that compared to SPF 10 participants, SPF 30 participants could stand higher daily doses of UVB without incurring sunburn. However, when a median daily UVB exposure of ~5 MEDs was reached, sunburn occurrence was the rule, irrespective of the SPF used.

The distribution of accumulated, or of daily UVA exposure did not correlate with differences observed for daily sunbathing duration or for in-study sunburn experience.

### DISCUSSION

This double blind, randomized trial conducted during summer 1998 confirmed the main findings of the trial conducted during summer 1997 (Autier et al, 1999). Quantities of sunscreen used were similar. Duration of sunbathing in both groups, as well as the difference in daily sunbathing duration between groups, were nearly equal to those observed in 1997. Earlier starting hour of sunbathing activities with use of the SPF 30 sunscreen was also achieved as well as similar sunburn experience in the two groups. However, because of restricted availability of dosimeters, the sample size in 1998 was smaller than in 1997. As a consequence, results obtained in 1998 had lower statistical significance than those obtained in 1997, and there was more instability in the data.

© 2000 Cancer Research Campaign 

**Table 2** Duration of sunbathing and exposure to UVB and UVA radiation

| | SPF 10[a] | SPF 30 | % Change | *P* value |
|---|---|---|---|---|
| **All participants** | | | | |
| Total number of days with sunbathing for all participants | 245 | 237 | – | – |
| Range | 1–20 | 3–16 | | |
| Hours of sunbathing per day with sunbathing | | | | |
| *All sunbathing sessions* | | | | |
| Median | 2.4 | 3.0[b] | + 25% | 0.054 |
| 1st–3rd quartile | 2.0–3.1 | 2.5–4.0 | | |
| *Sunbathing ≤ 6 PM* | | | | |
| Median | 2.1 | 2.8[c] | + 33% | 0.028 |
| 1st–3rd quartile | 1.6–3.1 | 2.5–3.8 | | |
| Number of days with sunbathing that ended ≥ 6 PM | 110 | 70 | – 34% | |
| % of total days with sunbathing | 45% | 29% | | < 0.01[b] |
| **Participants with informative dosimeters** | | | | |
| UVB (Joules/m$^2$) during sunbathing sessions with dosimeter | | | | |
| *Accumulated UVB exposure during holidays* | | | | |
| Median per volunteer | 5891 | 5863 | – 0.5 | 0.40 |
| Range | 273–16 829 | 2165–19 163 | | |
| *Median UVB exposure per day with sunbathing*[c] | | | | |
| Daily exposure | 841 | 984 | + 17 | 0.15 |
| 1st–3rd quartile | 532–1132 | 837–1192 | | |
| Daily exposure ≤ 6 PM | 781 | 984 | + 26 | 0.12 |
| 1st–3rd quartile | 483–1046 | 798–1187 | | |
| UVA (KJoules/m$^2$) during sunbathing sessions with dosimeter | | | | |
| *Accumulated UVA exposure during holidays* | | | | |
| Median per volunteer | 727 | 812 | + 11 | 0.70 |
| Range | 365–2399 | 274–3528 | | |
| *Median UVA exposure per day with sunbathing*[c] | | | | |
| Daily exposure | 136 | 125 | – 8 | 0.50 |
| 1st–3rd quartile | 89–176 | 93–187 | | |
| Daily exposure ≤ 6 PM | 135 | 124 | – 8 | 0.39 |
| 1st–3rd quartile | 87–167 | 90–186 | | |

[a] SPF: Sun Protection Factor; [b] $\chi^2$ test; [c] Denominator is the number of days with sunbathing during which the dosimeter was used.

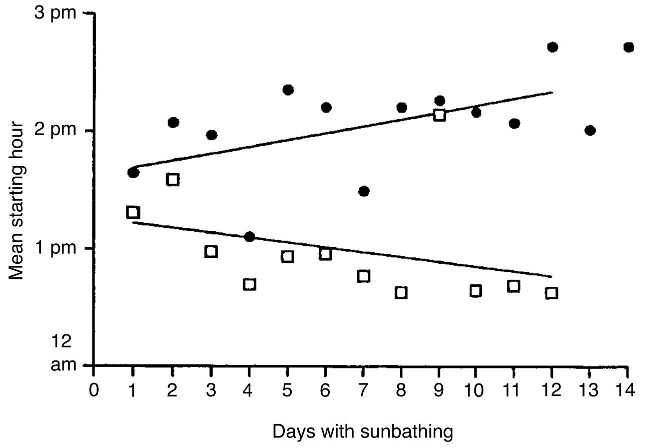

**Figure 1** Mean hour of start of sunbathing activities in days with sunbathing. Days without sunbathing were skipped. The time in the figure is the so-called 'summer hour' in Continental Europe, equivalent to the solar hour plus 2 hours. Blank squares represent SPF 30 participants; black circles represent SPF 10 participants. Linear regression lines have been drawn for each group using least square regression model. Student *t*-test for the difference in mean hours between the two groups: $t = 4.8$ with 24 degrees of freedom: $P < 0.001$

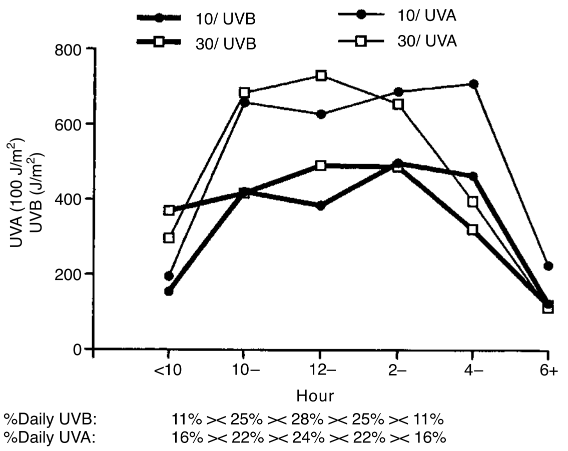

**Figure 2** Average daily doses of UVA and UVB received by participants during different periods of the day. The time in the figure is the so-called 'summer hour' in Continental Europe, equivalent to the solar hour plus 2 hours. The lower part of the figure shows typical proportions of UVA and UVB reaching earth's surface over a clear sky summer day in a Mediterranean area, at 40 degrees of Northern latitude (Diffey, 1991, adapted)

Some investigations have attempted to assess UV-light exposure of humans using biophysical methods (Dwyer et al, 1996; Diffey et al, 1996; Creech and Mayer, 1997), but to the best of our knowledge, this is the first study to assess UV exposure during sunbathing, an intermittent sun exposure pattern known to be associated with melanoma occurrence in Europe. In spite of the aforementioned limitations in statistical power, our results provide some insights into the relationship between intentional sun exposure and UV exposure.

We examined the extent to which technical aspects of the dosimeters or the way they were used could explain the results. Instructions given to participants for recording UV exposure

 *© 2000 Cancer Research Campaign*

**Table 3** Sunbathing and exposure to UVB and UVA radiation according to sunburn experience during holidays

| | SPF 10[a] | SPF 30 | % Change | *P* value |
|---|---|---|---|---|
| **All participants** | | | | |
| Median total number of hours of sunbathing ≤ 6 PM | | | | |
| No sunburn episode | 11.5 | 25.0 | + 174 | 0.06 |
| 1 sunburn episode | 18.5 | 17.0 | – 8 | 0.92 |
| ≥ 2 sunburn episodes | 17.0 | 19.0 | + 11 | 0.89 |
| Median total number of hours of sunbathing > 6 PM | | | | |
| No sunburn episode | 2.0 | 0.0 | – 200 | 0.04 |
| 1 sunburn episode | 1.5 | 1.0 | – 33 | 0.96 |
| ≥ 2 sunburn episodes | 2.0 | 1.0 | – 50 | 0.30 |
| Median number of hours of sunbathing per day with sunbathing | | | | |
| No sunburn episode | 2.1 | 3.0 | + 43 | 0.026 |
| 1 sunburn episode | 2.8 | 3.2 | + 14 | 0.41 |
| ≥ 2 sunburn episodes | 3.0 | 3.0 | 0 | 0.62 |
| Median number of hours of sunbathing ≤ 6 PM per day with sunbathing | | | | |
| No sunburn episode | 1.9 | 2.7 | + 42 | < 0.01 |
| 1 sunburn episode | 2.6 | 2.8 | + 8 | 0.59 |
| ≥ 2 sunburn episodes | 3.0 | 3.0 | 0 | 0.36 |
| **Participants with informative dosimeter** | | | | |
| UVB during sunbathing sessions ≤ 6 PM | | | | |
| *Median accumulated UVB during sunbathing (Joules/m$^2$)* | | | | |
| No sunburn episode | 3896 | 5620 | + 44 | 0.23 |
| 1 sunburn episode | 5473 | 3858 | – 30 | 0.69 |
| ≥ 2 sunburn episodes | 5633 | 6856 | + 22 | 0.71 |
| *Median UVB exposure per day with sunbathing (Joules/m$^2$)*[b] | | | | |
| No sunburn episode | 511 | 889 | + 74 | 0.08 |
| 1 sunburn episode | 828 | 1095 | + 32 | 0.41 |
| ≥ 2 sunburn episodes | 1046 | 1102 | + 5 | 0.58 |
| UVA during sunbathing sessions ≤ 6 PM | | | | |
| *Median accumulated UVA during sunbathing (KJoules/m$^2$)* | | | | |
| No sunburn episode | 718 | 739 | + 3 | 0.77 |
| 1 sunburn episode | 727 | 587 | – 19 | 0.81 |
| ≥ 2 sunburn episodes | 1489 | 1060 | – 29 | 0.58 |
| *Median UVA exposure per day with sunbathing (KJoules/m$^2$)*[b] | | | | |
| No sunburn episode | 90 | 108 | + 20 | 0.85 |
| 1 sunburn episode | 141 | 104 | – 26 | 0.81 |
| ≥ 2 sunburn episodes | 140 | 163 | + 16 | 0.47 |

[a] SPF: Sun Protection Factor; [b] Denominator is the number of days with sunbathing during which the dosimeter was used during sunbathing sessions mimicked the measure of horizontal solar irradiance commonly utilized for producing the 'solar UV-index' (ICNIRP, 1995; Geller et al, 1997; Jendritzky et al, 1997). As the UVA sensor had a smaller aperture angle than the UVB sensor, when the dosimeter veered off the sun, the UVA signal could drop more quickly than the UVB signal. This effect was normally more prevalent before 10 am or after 6 pm when the sun is closer to the horizon. Data in Figure 2 may suggest the presence of that angular effect early in the morning and late in the afternoon. However, there is no reason to believe that this angular effect was not equally distributed among the two randomization groups.

The difference in daily sunbathing duration correlated with differences in daily UVB exposure, but not with UVA or UVB exposure accumulated over all sunbathing sessions, or with daily UVA exposure. UVA irradiation throughout the day is more constant than UVB irradiation, and the UVB-to-UVA ratio in the solar spectrum is maximal around noon (IARC, 1992), i.e., around 2 pm in this trial (Figure 2). Results from this trial and from the 1997 trial (Autier et al, 1999) suggest that use of the SPF 30 sunscreen encouraged participants to sunbathe when UVB irradiation was maximal. In contrast, SPF 10 participants seemed keener to sunbathe later in the afternoon, when sunlight was less intense, that is, less rich in UVB radiation. Hence, proportionally to daily UVA exposure, SPF 30 participants were exposed to greater amounts of daily UVB than SPF 10 participants.

The largest differences between the two groups with regards to total number of sunbathing hours on holiday, daily sunbathing duration, and daily UVB exposure, were observed in participants who did not suffer from sunburn. Although only of borderline statistical significance, the positive interaction found between in-trial sunburn experience and randomization group suggested that absence of sunburn due to sunscreen use encourages extension of intentional sun exposure.

Sunburn is an inflammatory process of the skin essentially induced by UVB radiation. The 'sunburn threshold' (the quantity of UV in one day needed to trigger a sunburn) is lowest in subjects highly sun sensitive, and highest in subjects who never burn and tan easily when in the sun. In this trial, and in the 1997 trial (Autier et al, 1999), participants in both groups had similar sunburn experience during their holidays. This finding suggests that the UV delivered to the skin below the sunscreen layer was similar in both groups. The two groups differed in the way the daily UV doses were delivered to the skin. Sunscreen use during sunbathing would set the individual sunburn threshold at a higher level. Because of the longer time needed to reach the higher sunburn threshold, increase in time spent in sunbathing activities was possible, especially if no sunburn occurred. Bearing in mind that the greatest difference in melanoma risk or nevi count between sunscreen users and non-users was found in subjects free of sunburn history (Autier et al, 1995, 1998; Westerdahl et al, 2000), our results suggest that the association between sunscreen use and melanoma

*© 2000 Cancer Research Campaign*

would proceed from the ability of sunscreens to allow the repetition over short periods of exposures to UV doses below the sunburn threshold. This interpretation is consistent with laboratory data showing that a given UV dose delivered in multiple irradiation sessions (dose fractionation) or over a longer period of time (dose attenuation) is more efficient in causing skin tumours in mice (Forbes; 1981; Kelfkens et al, 1991).

### ACKNOWLEDGEMENTS

Supported by grants from the European Melanoma Group (Brussels, Belgium), and from the Europe against Cancer Programme of the European Commission. Neither the European Commission nor any person acting on its behalf is liable for any use made of the content of this article. The personal ultraviolet A and B dosimeters (pUVD) used in this study, and the software installed on them, are patent products of the European Melanoma Group, a.s.b.l., v.z.w. (Brussels, Belgium). The pUVDs have been engineered and produced by the JEG Join Engineering Group, S.C. (Brussels, Belgium).

### REFERENCES

Autier P (2000) Sunscreen and melanoma revisited. *Arch Dermatol* **136**: 423

Autier P, Doré JF, Lejeune FJ, Koelmel KF, Geffeler O, Hille P et al. (1994) Risk factors of the cutaneous melanoma: Results from an EORTC case-control study. *Melanoma Research* **4**: 79–85

Autier P, Doré JF, Schifflers E et al. (1995) Melanoma and use of sunscreens: an EORTC case-control study in Germany, Belgium and France. *Int J Cancer* **61**: 749–755

Autier P, Doré JF, Cattaruzza MS, Renard F, Luther H, Gentiloni-Silverj F et al. for the EORTC Melanoma Group (1998) Sunscreen use, wearing clothes and nevi number in 6- to 7-year-old European children. *J Nat Cancer Inst* **90**: 1873–1881

Autier P, Doré JF, Négrier S, Liénard D, Panizzon R, Lejeune FJ, Guggisberg D and Eggermont AMM (1999) Sunscreen use and duration of sun exposure: a double blind randomized clinical trial. *J Natl Cancer Inst* **91**: 1304–1309

Bigby M (1999) The sunscreen and melanoma controversy. *Arch Dermatol* **135**: 1526–1527

Creech LL and Mayer JA (1997) Ultraviolet radiation exposure in children: a review of measurement strategies. *Ann Behav Med* **19**: 399–407

Diffey BL (1991) Solar ultraviolet radiation effects on biological systems. *Phys Med Biol* **36**: 299–328

Diffey BL, Gibson CJ, Haylock R and McKinlay AF (1996) Outdoor ultraviolet exposure of children and adolescents. *Br J Dermatol* **134**: 1030–1034

Donawho C and Wolf P (1996) Sunburn, sunscreen, and melanoma. *Current Opinion Oncol* **8**: 159–166

Dwyer T, Blizzard L, Gies PH, Ashbolt R and Roy C (1996) Assessment of habitual sun exposure in adolescents via questionnaire – a comparison with objective measurement using polysulphone badges. *Mel Research* **6**: 231–239

Elwood JM and Gallagher RP (1998) Body distribution of cutaneous malignant melanoma in relationship to patterns of sun exposure. *Int J Cancer* **78**: 276–280

Elwood M and Jopson J (1997) Melanoma and sun exposure: an overview of published studies. *Int J Cancer* **73**: 198–203

Fitzpatrick TB (1998) The validity and practicability of sun-reactive skin types I through VI. *Arch Dermatol* **124**: 869–871

Forbes PD (1981) Photocarcinogenesis: an overview. *J Invest Dermatol* **77**: 139–148

Geller AC, Hufford D, Miller DR, Sun T, Wyatt SW, Reilley B et al. (1997) Evaluation of the ultraviolet index: Media reactions and public response. *J Am Acad Dermatol* **37**: 935–941

IARC (1992) International Agency for Research on Cancer. Solar and ultraviolet radiation. Monographs on the evaluation of the carcinogenic risk of chemicals to humans. *IARC*, Lyon, **Vol 55**: 285–290

International Commission on Non-Ionizing Radiation Protection. (1995) Global Solar UV index. Publication ICNIRP-**1/95**, Oberschleissheim, Germany

Jendritzky G, Staiger H and Bucher K (1997) UV prognosis and UV index services in Europe. In: *Skin Cancer and UV radiation*. Altmeyer P, Hoffmann K, Stücker M (eds). Springer, Berlin, pp 37–49

Kelfkens G, van Weelden H, de Gruijl F and van der Leun JC (1991) The influence of dose rate on ultraviolet tumorigenesis. *J Photochem Photobiol B Biol* **10**: 41–50

Lui H, Gallagher RP and McLean DI (1997) Use and misuse of sunscreens. In: *Skin cancer and UV radiation*. Altmeyer P, Hoffmann K, Stucker M (eds). Springer-Verlag, Berlin, pp 333–342

Osterlind A, Tucker MA, Stone BJ and Jensen OM (1988) The Danish case-control study of cutaneous malignant melanoma. II. Importance of UV-light exposure. *Int J Cancer* **42**: 319–324

Setlow RB and Woodhead AD (1994) Temporal changes in the incidence of malignant melanoma: explanation from action spectra. *Mutation Research* **307**: 365–374

Westerdahl J, Ingvar C, Masback A and Olsson H (2000) Sunscreen use and malignant melanoma. *Int J Cancer* **87**: 145–150

*© 2000 Cancer Research Campaign*
